# Sleep-Induced Glottis Closure in Multiple System Atrophy Evaluated by Four-Dimensional Computed Tomography

**DOI:** 10.3389/fmed.2020.00132

**Published:** 2020-04-17

**Authors:** Rumi Ueha, Eriko Maeda, Kenji Ino, Takahiro Shimizu, Taku Sato, Takao Goto, Tatsuya Yamasoba

**Affiliations:** ^1^Department of Otolaryngology, The University of Tokyo, Tokyo, Japan; ^2^Department of Computational Diagnostic Radiology and Preventive Medicine, The University of Tokyo Hospital, Tokyo, Japan; ^3^Imaging Center, The University of Tokyo Hospital, Tokyo, Japan; ^4^Department of Neurology, The University of Tokyo, Tokyo, Japan

**Keywords:** sleep-induced glottis closure, four-dimensional computed tomography, multiple system atrophy, sleep apnea, upper airway

## Abstract

Multiple system atrophy (MSA) is a progressive neurodegenerative disorder. Since patients with MSA often have sleep-related respiratory disorders including upper-airway obstruction and/or central sleep disturbance, appropriate evaluation of the upper airway especially during sleep may be indispensable. Fiberoptic laryngoscopy during diazepam-induced sleep has been reported for upper-airway obstruction verification. However, some patients cannot endure the uncomfortable sensation of the fiberscope. To address these issues, we devised a protocol of four four-dimensional computed tomography (4D-CT) for upper-airway evaluation during sleep. Here, we report the case of patient with MSA who was evaluated for upper-airway obstruction during sleep using 4D-CT. A 46-year-old man (height 1.60 m, weight 79 kg) was admitted to our neurological department for tracheal intubation because of a sudden onset of respiratory failure occurring at night. At the age of 45 years, he was diagnosed as MSA with predominant parkinsonism. As pulmonary disease had been excluded and his swallowing was normal, our differential diagnoses were central sleep apnea or obstructive sleep apnea related to his MSA or obstructive sleep apnea (SA) related to his obesity. A tracheostomy was done to maintain the airway after extubation. Polysomnography showed obstructive SA and not central SA. Awake fiberoptic laryngoscopy showed no upper airway obstruction but bilateral vocal abduction impairment (BVAI) during inspiration. To assess the spatial and temporal conditions of the upper respiratory tract—the patient could not tolerate sleep laryngoscopy—we carried out a 4D-CT. Reconstructed 4D-CT images of respiration during sleep showed clear abnormalities: glottis closure at the terminal stage of inspiration and subsequent velopharyngeal closure. As glottis closure does not occur normally in obesity patients, the cause of the respiratory failure in this patient was considered MSA-related sleep-induced airway obstruction. We decided to keep the tracheostoma, because BVAI in patients with MSA may be getting worse, although central apnea after tracheostomy may cause sudden central origin-related death; 4 months postoperatively, the patient had experienced no further airway-related complications. This report indicates that 4D-CT sequential upper-airway assessment during sleep is useful for determining the abnormalities causing obstructive SA in patients with MSA.

## Background

Multiple system atrophy (MSA) is a progressive neurodegenerative disorder that causes systemic autonomic dysfunction, parkinsonian disorder, and cerebellar dysfunction in various combinations. MSA is generally divided into the parkinsonian (MSA-P) and cerebellar (MSA-C) variant types (1).

Patients with MSA often have sleep-related respiratory disorders including inspiratory stridor (a high-pitched sound) and sleep apnea (SA) (2); 34% of such patients have inspiratory stridor and 4% have respiratory stridor as the initial symptom of MSA (3). SA develops a mean of 5.8 years after MSA onset, and its causes include upper-airway obstruction and/or central sleep disturbance (3). Upper-airway obstruction can be caused by bilateral vocal fold motion impairment (VFMI), floppy epiglottis, and floppy arytenoid and can cause sudden death. Thus, appropriate evaluation of the upper airway especially during sleep may be indispensable, and tracheostomy should be performed as needed based on the grade of upper-airway obstruction (4, 5).

Flexible laryngeal fibroscopy is commonly used for upper-airway evaluation, and the use of fiberoptic laryngoscopy during diazepam-induced sleep has been reported for upper-airway obstruction verification (6). However, some patients cannot endure the uncomfortable sensation of the fiberscope indwelled in the upper airway, and the recommended diazepam dose does not induce sleep to all patients, rendering fibroscopic examination impractical. To address these issues, we devised a protocol of four four-dimensional computed tomography (4D-CT) for upper-airway evaluation during sleep. 4D-CT is a new method of three-dimensional computed tomography, depicting moving structures by creating a dynamic-volume dataset over a period of time and has been widely researched and used in cardiology and pulmonology (7, 8). Here, we describe our method of asleep upper-airway evaluation using 4D-CT and the case of patient with MSA who was evaluated for upper-airway obstruction during sleep using 4D-CT.

## Case Presentation

A 46-year-old man (160 cm, 79 kg, body mass index: 30.9 kg/m^2^) was emergently admitted to the neurological department of our institution with tracheal intubation because of sudden respiratory failure at night. He had no congenital malformations or developmental, cardiovascular, respiratory, or endocrine disorders. At age 38 years, a high-pitched snore was noticed by his partner. When he was ~44 years old, he began complaining of neck and trunk rigidity, bradykinesia, postural tremor, and dysuria. At age 45 years, he was diagnosed with MSA with predominant parkinsonism.

As pulmonary inflammatory diseases were excluded by serologic and radiological examinations and swallowing function was substantially normal, we considered the possibility of MSA-related central respiratory disorder or obstructive respiratory disorder due to MSA and/or obesity. Tracheostomy was performed to maintain the airway after extubation, although continuous positive airway pressure may have also been helpful. At that time, physical examination revealed his temperature to be 36.5 degree Celsius, heart rate 77 bpm, respiratory rate 24, BP 108/81, and O2 saturation 95% on room air. Blood gas analysis revealed a pH of 7.40, carbon dioxide partial pressure of 33.8 mmHg, and oxygen partial pressure of 74.7 mmHg. Polysomnography showed obstructive SA (OSA), but not central SA. Awake fiberoptic laryngoscopy displayed no upper-airway obstruction but slight vocal-cord abduction impairment during inspiration, although the patient could endure the laryngoscope for <1 min because of discomfort. Sleep laryngoscopy was considered for upper-airway evaluation, although it cannot simultaneously evaluate different sites, but the patient refused because of discomfort. To assess the spatial and temporal conditions of the upper respiratory tract, including the nasal passages, pharynx, larynx, and portion of the trachea, we performed 4D-CT during sleep. The effective dose was 1.84 mSv (CT dose index: 19.5 mGy, dose–length product: 311 mGy.cm).

**Figure 1:**
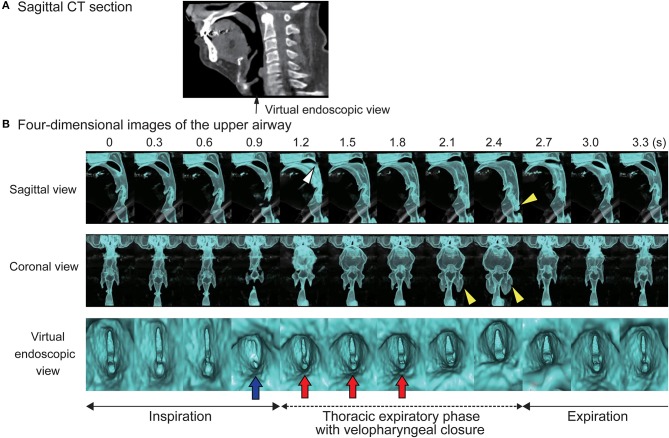
Four-dimensional computed tomographic (4D-CT) images. **(A)** Sagittal section of computed tomography. **(B)** Serial four-dimensional computed tomographic images of upper airway during sleep shows glottis closure at a terminal stage of inspiration (blue arrow), subsequent velopharyngeal closure (white arrow head), expansion of the pharyngeal cavity (yellow arrow heads), and succeeding glottis opening with increase of pharyngeal pressure (red arrows).

**Figure 2:**
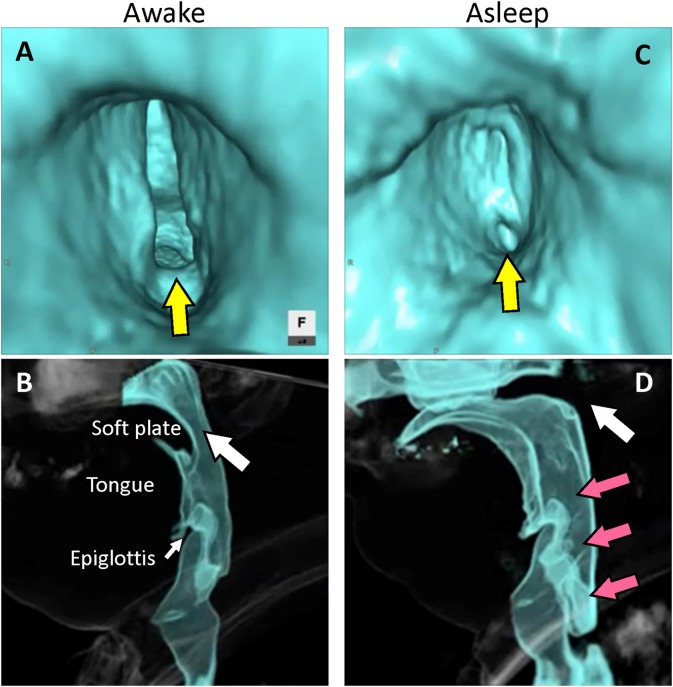
Reconstructed images of four-dimensional computed tomography. **(A)** Virtual endoscopic image from the subglottic view. The glottis remains open at the terminal stage of inspiration during wakefulness (yellow arrow). **(B)** Lateral view. The velopharyngeal space remains open at the initial stage of exhalation during wakefulness (white arrow). **(C)** Virtual endoscopic image from the subglottic view. The glottis closes at the terminal stage of inspiration during sleep (yellow arrow). **(D)** Lateral view. The soft plate becomes elevated and velopharyngeal closure occurs at the initial stage of exhalation during sleep (white arrow) with expansion of the pharyngeal space (red arrows).

We placed the patient in the supine position, sedated him with intravenous administration of 5 mg diazepam, and closed the tracheostoma with adhesive tape. Reconstructed 4D-CT images of respiration during sleep (Figures 1A,B; Videos 1, 2) revealed abnormal upper-airway findings compared with the images acquired during awake respiration: glottis closure at the terminal stage of inspiration (normally, the glottis opens during inspiration) (Video 3), subsequent velopharyngeal closure (normally, the velopharyngeal space is open during nasal respiration), and succeeding glottis opening and expansion of pharyngeal pressure, but no other upper airway-obstruction findings, such as glossoptosis, pharyngeal-cavity narrowing, and retrodisplaced epiglottis (Figures 2A–D).

Thus, MSA-related sleep-induced airway obstruction was considered the cause of the respiratory failure. Consequently, retaining the tracheostoma was determined to be indispensable, although central apnea after tracheostomy may cause sudden central origin-related death; 14 months postoperatively, the patient had experienced no further airway-related complications.

## Diagnostic Tests: Image Acquisition of 4D-CT

CT scanning was performed using second-generation 320-row CT (Aquilion ONE Vision Edition; Canon Medical Systems Corporation, Otawara, Japan). Prior to 4D-CT scanning, a volume scan of the face and neck was performed to verify that the uvula and glottis were included within the 4D volume. 4D-CT was performed with the following parameters: detector configuration, 0.5 × 320 mm; tube voltage, 120 kVp; tube current, 20 mA; scan length, 16 cm; scan duration, 4.4 s; and no gantry tilt. The reconstructed slice thickness and increment were 0.50 mm. Images were reconstructed using a medium soft tissue kernel (FC04) with an iterative reconstruction algorithm (AIDR 3D enhanced mild). For processing, images were transferred to a workstation (ZIO Station System 2; Ziosoft, Tokyo, Japan) and reconstructed at ~100 ms intervals. This study was approved by the Human Ethics Committee of the University of Tokyo (No. 2487). Written informed consent was obtained from the patient.

## Discussion

Here, we reported on the usefulness of 4D-CT for upper-airway assessment in patients with MSA in a state of sleep over a period of time. MSA is a progressive neurodegenerative disorder for which autonomic dysfunction, parkinsonian disorder, and cerebellar dysfunction present as main symptoms. Apart from other symptoms such as respiratory disorder and dysphagia, confirmation of both autonomic dysfunction and movement impairment may inform the diagnosis of MSA (1, 9).

Respiratory stridor, SA, and respiratory insufficiency are part of the clinical spectrum of MSA (10, 11). Respiratory stridor can be caused by floppy epiglottis, floppy arytenoid, and VFMI (4, 5). Two plausible mechanisms for VFMI-related stridor have been suggested: (1) severe loss of neurons in the nucleus ambiguous resulting in hypoactivity of the laryngeal abductor and neurogenic atrophy in the posterior cricoarytenoid muscle and (2) dystonia induced by hyperactivity of the laryngeal adductor muscles (3, 4).

SA is divided into two types: obstructive and central, respectively, caused by upper-airway obstruction and degeneration of the sleep centers in the brain (1, 2, 12). Sudden death is common in patients with MSA and usually occurs during sleep (3, 13), but it is unclear whether the sudden death results from VFMI and/or central hypoventilation or from SA or other causes. OSA may be partly caused by laryngeal constriction, including VFMI. Considering that patients with MSA-P developed VFMI in higher frequency and earlier after MSA onset than did patients with MSA-C (under submission), vocal fold movement should be monitored especially in patients with MSA-P during sleep. As this case was of a patient with MSA-P with sudden respiratory failure at night, differentiation of SA (OSA or central SA) and laryngeal evaluation during sleep were indispensable to decide on the course of treatment. After polysomnography, which revealed OSA but not central SA, we assessed upper-airway obstruction during sleep using 4D-CT instead of direct laryngoscopy. Continuous positive air pressure has been reported to be effective for eliminating nocturnal stridor in MSA patients with vocal fold movement restriction (14), although nasal positive pressure hyperventilation was reported to narrow the glottis (15). Accordingly, it is difficult to determine the application timing of non-invasive positive pressure ventilation for patients with MSA.

4D-CT can be conducted at a position similar to that normally assumed during sleep and does not involve uncomfortable sensations, in contrast to laryngeal fibroscopy placed into the nasopharynx. As 4D-CT can simultaneously evaluate different sites including the velopharynx, oropharynx, hypopharynx, larynx, and portion of the trachea, even during sleep, it might be more useful for spatial and temporal assessment of the upper respiratory tract during sleep compared to direct laryngoscopy. Magnetic resonance imaging (MRI) can also provide detailed information of the airway during respiration, but is limited to only single-slice cines (16). Considering the advantageous features of 4D-CT such as the short examination duration, higher spatial and temporal resolution, and quieter environment, 4D-CT could be preferable to MRI in evaluation of the airway during sleep, although radiation-exposure must be considered. The effective dose in our 4D-CT photographing conditions (1.84 mSv) is higher than that of another study that used the same photographing apparatus (average effective dose: 0.24 mSv, examination duration: 2.5 s) (16), but seems acceptable in comparison with those of other studies (1.89–3.41 mSv) (17, 18). Further, the 4D-CT technique also has the following potential limitations: ionizing radiation exposure, limitation to the supine position, the confounding effect of diazepam, the requirement to track inspiration and expiration during the scans, the necessity of CT image reconstruction, and the technique is not available in all facilities.

In conclusion, in the present report we indicate that 4D-CT sequential upper-airway assessment during sleep might be useful for assessing the potential contributors causing OSA in patients with MSA. This technique should be reserved for patients with suspected OSA who cannot tolerate sleep laryngoscopy.

## Data Availability Statement

The datasets generated for this study are available on request to the corresponding author.

## Ethics Statement

Written informed consent was obtained from the individual(s) for the publication of any potentially identifiable images or data included in this article.

## Author Contributions

We all cared for the patient. RU, EM, and KI performed radiological examinations. RU was responsible for writing the manuscript and collecting data. TY contributed to the critical review. All authors contributed to the writing of the report. Written consent for publication was obtained from the patient.

## Conflict of Interest

The authors declare that the research was conducted in the absence of any commercial or financial relationships that could be construed as a potential conflict of interest.

## Supplementary Material

Video 1 Anterior-posterior view of the airway. Both vocal folds normally remain open during respiration, and move apart during inspiration. The area of the hypopharyngeal space does not change during respiration. However, in this patient, the vocal folds abnormally close during sleep at the terminal stage of inspiration. Subsequently, after expansion of the pharyngeal cavity at the initial stage of exhalation, exhalation can be carried out.

Video 2 Lateral view of the airway Under nasal respiration, the soft plate normally does not change its position, and velopharyngeal closure does not occur during breathing. However, in this patient, the soft palate becomes abnormally elevated during sleep at the initial stage of exhalation, followed by expansion of the pharyngeal space. With release of the abnormal velopharyngeal closure, the patient can exhale smoothly.

Video 3 Virtual endoscopic view from the subglottis. Both vocal folds normally open and maintain the airway during respiration. However, in this patient, the bilateral vocal folds abnormally adduct and close at the terminal stage of inspiration, while expiratory abduction movement of the vocal folds is conserved.
